# Value-added Synthesis of Graphene: Recycling Industrial Carbon Waste into Electrodes for High-Performance Electronic Devices

**DOI:** 10.1038/srep16710

**Published:** 2015-11-16

**Authors:** Hong-Kyu Seo, Tae-Sik Kim, Chibeom Park, Wentao Xu, Kangkyun Baek, Sang-Hoon Bae, Jong-Hyun Ahn, Kimoon Kim, Hee Cheul Choi, Tae-Woo Lee

**Affiliations:** 1 Department of Materials Science and Engineering, Pohang University of Science and Technology (POSTECH), Pohang, Gyungbuk 790-784, Republic of Korea; 2 Department of Chemistry, Pohang University of Science and Technology (POSTECH), Pohang, Gyungbuk 790-784, Republic of Korea; 3 Center for Artificial Low Dimensional Electronic Systems (CALDES), Institute for Basic Science (IBS), Pohang, Gyungbuk 790-784, Republic of Korea; 4 Center for Self-assembly and Complexity (CSC), Institute for Basic Science (IBS), Pohang, Gyungbuk 790-784, Republic of Korea; 5 School of Electrical and Electronic Engineering, Yonsei University, Seoul 120-749, Republic of Korea; 6 Division of Advanced Materials Science, Pohang University of Science and Technology (POSTECH), Pohang, Gyungbuk 790-784, Republic of Korea

## Abstract

We have developed a simple, scalable, transfer-free, ecologically sustainable, value-added method to convert inexpensive coal tar pitch to patterned graphene films directly on device substrates. The method, which does not require an additional transfer process, enables direct growth of graphene films on device substrates in large area. To demonstrate the practical applications of the graphene films, we used the patterned graphene grown on a dielectric substrate directly as electrodes of bottom-contact pentacene field-effect transistors (max. field effect mobility ~0.36 cm^2^·V^−1^·s^−1^), without using any physical transfer process. This use of a chemical waste product as a solid carbon source instead of commonly used explosive hydrocarbon gas sources for graphene synthesis has the dual benefits of converting the waste to a valuable product, and reducing pollution.

Graphene is a two-dimensional monolayer of carbon atoms that has outstanding mechanical, chemical and electrical properties^1^,^2^,^3^,^4^,^5^,^6^. To exploit these properties, various graphene synthesis methods have been suggested^7^,^8^,^9^,^10^,^11^,^12^,^13^,^14^,^15^,^16^,^17^,^18^,^19^,^20^,^21^,^22^,^23^,^24^,^25^,^26^,^27^,^28^,^29^,^30^,^31^,^32^,^33^,^34^,^35^,^36^,^37^,^38^,^39^,^40^,^41^,^42^,^43^,^44^,^45^,^46^,^47^ since it was first detached from graphite by mechanical exfoliation^6^. Among existing methods to obtain large, high-quality graphene layers, epitaxial approaches use substrates such as SiC to guide direct growth of graphene^7^,^8^,^9^,^10^. Some of these methods can provide large-scale graphene, but it is difficult to detach from the substrates and the cost of substrates is relatively high. Chemical vapor deposition (CVD) using CH_4_ or C_2_H_2_ gas has been used to produce a large-area and highly-functional graphene on a catalytic metal, and is regarded as the most promising graphene synthesis method^25^,^26^,^27^,^28^,^29^,^30^,^31^,^32^,^33^,^34^,^35^,^36^,^37^,^38^. However, CVD requires an additional process to transfer the graphene films onto dielectric substrates that are required in electronic devices. Moreover, CVD uses flammable and explosive hydrocarbon gases. Although methods to grow graphene growth from solid-carbon sources such as polymers, organic, and amorphous carbon have been reported^39^,^40^,^41^,^42^, they also require an additional physical step to transfer the graphene to the target device substrates. Development of methods to that use solid sources of carbon and that grow patterned graphene films directly on device substrates without using explosive hydrocarbon gases and physical transfer^43^,^44^,^45^,^46^ is of prime importance from the perspective of sustainable industrial mass production. However, previous studies of graphene growth from solid-carbon sources have not fully shown the utillization of graphene as electrodes for electronic devices. In addition, graphene growth from chemical by-product, waste or residue would have additional environmental benefits^47^.

Here we introduce a simple, scalable, transfer-free, ecologically sustainable, value-added method to synthesize patterned graphene electrodes for high-performance electronic devices by recycling coal tar pitch (CTP) as an inexpensive carbon feedstock. CTP as an industrial by-product of steel production and is mainly composed of a complex mixture of aromatic hydrocarbons and heterocyclics^48^. Every year, steelworks produce a huge amount of CTP, which is toxic and causes severe environmental problems^49^. Recycling of this low-cost waste into high-added-value materials such as graphene could contribute greatly to environmental cleanliness and sustainable production of graphene. CTP solution was spin-coated on SiO_2_/Si substrates as a carbon feedstock. The CTP film was passivated with a Ni capping layer, then annealed under high temperature, low vacuum and a reducing atmosphere. The method produces multi-layer graphene on the top and bottom surfaces of the Ni layer. This result suggests that carbon atoms are released during pyrolysis of the CTP, then diffuse into the Ni layer during annealing, and precipitate to form graphene on both sides of the Ni layer upon cooling^43^,^44^,^45^,^46^,^50^. The Ni capping layer on a carbon source functions both as a passivation layer on the evaporable CTP film under the growth temperature and as a metal catalyst for efficient graphene growth^43^,^45^,^46^. Although we can obtain graphene on both sides of the Ni layer at the same time, to exploit the fact that we can fabricate graphene on substrate directly without a transfer process, we used graphene formed between the Ni capping layer and SiO_2_/Si substrate in an electronic device. We demonstrated an organic field-effect transistor (OFET) as the first example of organic electronic devices using graphene electrodes based on direct patterned growth from solid carbon source on the device substrate without using any additional graphene-transfer process; the Ni capping layer was removed by wet etching, thereby leaving the graphene films on SiO_2_/Si substrate.

## Results

### The growth of CTP-derived graphene and its characterization

**Figure 1:**
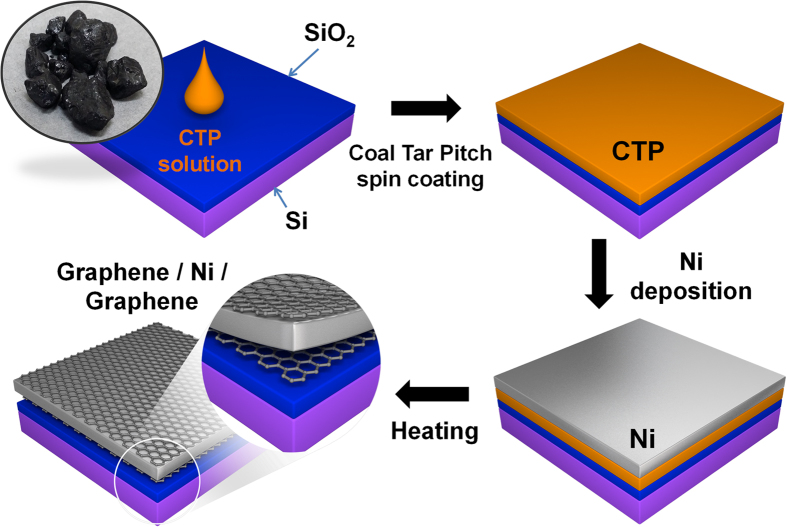
Schematic of basic synthesis procedure. Graphene films are converted from coal tar pitch on the top and bottom surface of Ni layer at 1100 °C for 4 min under low vacuum and a reducing atmosphere.

**Figure 2:**
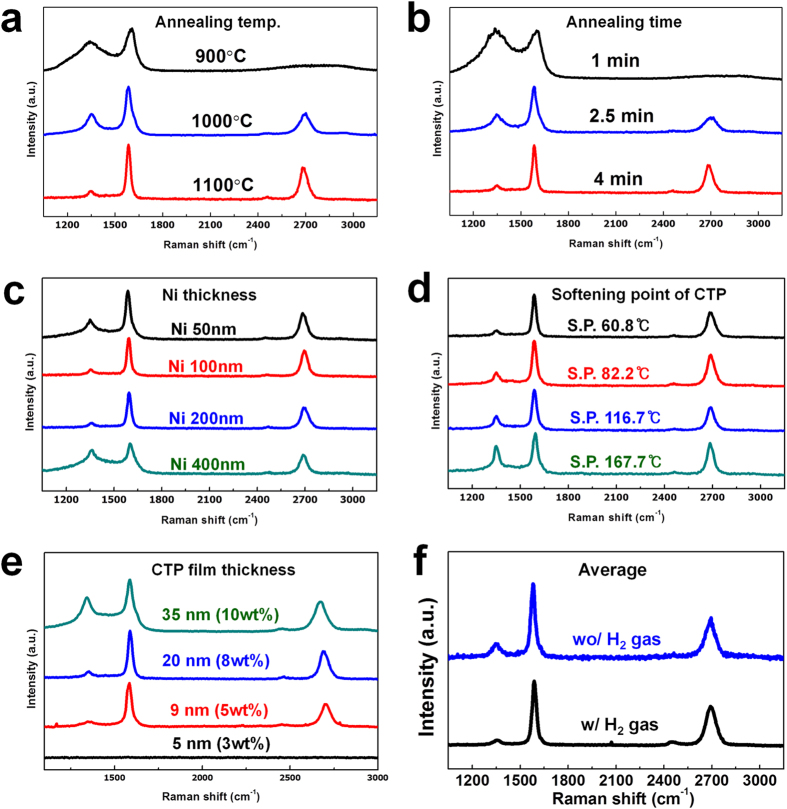
Raman spectra of coal tar pitch-derived graphene grown under Ni layer after annealing. (**a**) Raman spectra of graphene depending on the annealing temperature for 4 min. (**b**) Raman spectra of graphene depending on the annealing time at 1100 °C. (**c**) Raman spectra of graphene depending on Ni layer thickness at 1100 °C for 4 min. (**d**) Raman spectra of graphene depending on the softening point of coal tar pitch at 1100 °C for 4 min. (**e**) Raman spectra of graphene depending on the concentration of CTP solution at 1100 °C for 4 min. (**f**) Raman spectra of graphene grown with and without H_2_ gas. Lines have been shifted vertically for clarity.

In the basic graphene growth process (Fig. 1), CTP (softening point 60.8 °C) was dissolved (8 wt%) in quinoline solvent. Before spin-coating, SiO_2_ (500 nm)/Si substrates (2 cm × 2 cm) were treated with UV/ozone for 30 min to establish good wetting between CTP solution and substrate, and then a 20-nm thickness of CTP film was deposited on SiO_2_/Si substrates uniformly by spin coating (6000 rpm, 60 s). The thickness of CTP film was measured using ellipsometry and the film uniformity was confirmed using atomic force microscopy (AFM) (Table S1). A 200-nm-thick Ni metal-capping layer was deposited on top of the CTP film to prevent it from vaporizing and for use as a metal catalyst layer during annealing. The samples were then thermally annealed in a furnace under Ar (50 sccm) and H_2_ (10 sccm) gas, and low vacuum (~330 mTorr) at 900–1100 °C for 1–4 min to find the optimal conditions for graphene synthesis. After annealing, the samples were cooled to room temperature under the same Ar/H_2_ flow. A Raman spectroscopy system with excitation of 532 nm was used to evaluate whether CTP films had turned into graphene films^51^,^52^. To confirm the quality of graphene films formed between Ni capping layer and SiO_2_/Si substrate in various growth conditions, the Ni layer and the graphene films grown on top of the Ni layer were removed by simple immersion in aqueous FeCl_3_ solution for 1 min; the underlying graphene films remained on the SiO_2_/Si substrate. This process enabled direct growth of graphene films on dielectric substrates, and avoided damage to the graphene that could occur if the graphene were transferred using additional transfer process. In the average Raman spectrum (n = 2500 points), the D peak (~1350 cm^−1^) corresponds to defects in the graphene films. At annealing time of 4 min, the D peak intensity decreased as annealing temperature increased in the range of 900–1100 °C (Fig. 2a). When annealing temperature was 1100 °C, the D peak decreased as annealing time increased from 1 to 4 min (Fig. 2b). The temperature dependence of diffusion is given by:


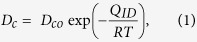


where *D*_c_ is diffusion coefficient, *D*_co_ is the maximum diffusion coefficient at infinite temperature and *Q*_ID_ is the activation enthalpy for the diffusion, *R* is the gas constant and *T* is absolute temperature. This equation is Fick’s first law of diffusion expressed in terms of an Arrhenius type. Based on this equation, increase in annealing temperature increases the amount of carbon that can dissolve in the Ni film, and therefore increases the amount of carbon precipitated to the Ni surface upon cooling, and increase in annealing time gives sufficient supply of carbon atom to form high-quality graphene at high temperature^41^. The Ni film thickness significantly affected the quality of graphene (Fig. 2c). Among various thicknesses of Ni film tested, a 200-nm-thick layer showed the smallest D peak in the Raman spectrum. When the thickness of the Ni layer was decreased to 50 nm, it vaporized and agglomerated at high temperature, so it cannot play a role as an effective catalyst owing to its smaller coverage on the CTP film and the resulting graphene was more defective. A 400-nm-thick Ni layer was too thick to allow the diffused carbon atoms to precipitate entirely from the Ni bulk on to the surface during cooling, so the Raman spectrum showed a large D peak^50^.

The effects of various softening points (SPs) and concentrations of CTP solution were investigated, because these factors also affect the graphene quality. As the SP of CTP increased (due to an increase in the portion of large molecules in CTP solution), the film thickness increased at the same concentration (8 wt%) and spin coating condition (6000 rpm, 60 s) (Table S1). The number of defects in the graphene increased with SP due to increase of thickness; the remaining hydrocarbon sources, pyrolized from CTP molecules, which did not dissolve into the Ni layer at high temperature due to the solubility limit of Ni layer was converted to amorphous carbon and that their residual amorphous carbon can increase D peak (Fig. 2d). The effect of different CTP film thickness varied with concentration of CTP solution (SP 60.8 °C) (Fig. 2e). Raman spectra suggest that the optimum concentration was 8 wt% (20-nm-thick CTP film), but thicker (35 nm) CTP films from 10 wt% solution have a relatively high D peak that might be caused by residual carbon sources that result in formation of amorphous carbon. A 2 wt% CTP solution (5-nm-thick CTP film) formed a discontinuous graphene film due to a lack of carbon sources during annealing.

Furthermore, CTP-derived films on top of the Ni layer were transferred onto SiO_2_/Si substrates to measure the average Raman spectrum. We used a poly[methyl methacrylate] (PMMA)-layer-supported graphene transfer process with metal etching solution (FeCl_3_) described elsewhere^28^. From the Raman analysis of graphene grown on Ni layer (Figure S1), we confirmed that the optimum annealing condition was 1100 °C for 4 min (with Ni 200 nm, SP = 60.8 °C), which is the same as that of the graphene grown under a Ni layer. These results indicate that characteristics of graphene films on both sides of the Ni layer have the same tendency in the several growth conditions tested.

We also obtained graphene films from the growth process without using H_2_ gas. Graphene films formed even under low-vacuum condition with Ar atmosphere (~3.8 mTorr), although the average Raman spectrum had higher D peak intensity (Fig. 2e) than did the spectra of graphene fabricated in the presence of H_2_ gas. Because H_2_ gas assists the crystallization of graphene by removing defects in carbon such as dangling bonds, and by eliminaing certain impurities from the metal subustrate^53^, the absence of H_2_ gas induces defects in graphene. After H_2_-gas-free synthesis, Raman spectra from different points on the sample (Figure S2) indicate that of mono-layer to few-layer graphene had formed. This synthesis method does not use any explosive gases (CH_4_ and H_2_), and can be used to mass-produce graphene easily and safely.

**Figure 3:**
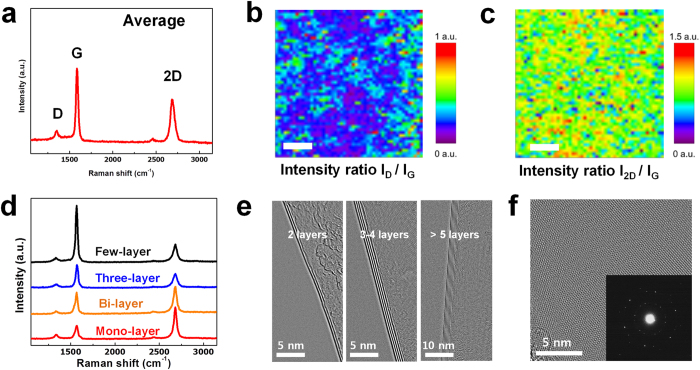
(a) Average Raman spectrum (2500 points) of coal tar pitch-derived graphene grown under Ni layer. (**b**) Raman mapping (100 × 100 μm) of D-to-G band peak intensity ratio (I_D_/I_G_) in coal tar pitch-derived graphene grown on Ni layer. Scale bar: 20 μm. (**c**) Raman mapping of 2D-to-G band peak intensity ratio (I_2D_/I_G_) in coal tar pitch-derived graphene grown on Ni layer. Scale bar: 20 μm. (**d**) Raman spectra of mono-layer to few-layer graphene formed on the SiO_2_/Si substrates that exists in the mapping area. (between Ni layer and SiO_2_/Si substrate). Lines have been shifted vertically for clarity. (**e**) TEM images of graphene films at the folded edge (**f**) TEM image of graphene surface. Inset: hexagonal electron diffraction pattern of graphene films.

CTP-derived graphene films grown on the SiO_2_/Si substrates at 1100 °C for 4 min were prepared for use in further characterization. This annealing condition was chosen based on the results of preliminary trials. In the Raman scanning over a large area (100 × 100 μm, 2500 points), pronounced peaks occurred at ~1580 cm^−1^ (G peak) and ~2700 cm^−1^ (2D peak), in addition to the D peak^51^,^52^. The high quality of graphene was demonstrated by the average Raman spectrum of all points over the scanned area (ratio of D peak intensity I_D_ to G peak intensity I_G_ ~0.1) (Fig. 3a). The quality of graphene was further confirmed by Raman mapping of I_D_/I_G_ (Fig. 3b). On most of the point I_D_/I_G_ < 0.2; this low value suggests that the graphene layer has few surface defects. The number of layers and the uniformity of graphene over this area were also illustrated by Raman mapping of the 2D-to-G peak intensity ratio (I_2D_/I_G_) (Fig. 3c). Raman mapping of I_2D_/I_G_ indicated that the multi-layer graphene consisted of mono-layer to few-layer portions (Fig. 3d), and that ~90% of the surface had I_2D_/I_G_ ~0.7, which is the signature of three-layer graphene. In the average Raman spectrum, the 2D peak had full width at half-maximum of ~58 cm^−1^ and I_2D_/I_G_ of ~0.62; these values are similar to those of three-layer graphene grown using CVD^25^,^28^. The CTP-derived multi-layer graphene was further characterized by transmission electron microscopy (TEM). The graphene films were separated from SiO_2_ layer using sodium hydroxide solution (1M), then transferred to the TEM grids. TEM images at the folded edge of graphene films show that the multilayer graphene consisted of mono-layer to few-layer graphene with regular interlayer spacing (~0.34 nm) (Figure S3) in agreement with our Raman analysis data (Fig. 3e). Hexagonal crystalline structure of graphene was observed on a randomly-imaged graphene surface, (Fig. 3f) and electron diffraction on the graphene films revealed hexagonal patterns which are typically observed in multilayer graphene films (Fig. 3f**, inset**)^26^,^45^. The measured sheet resistance of graphene grown on dielectric substrate was ~1 kΩ/sq and the minimum value was 906 Ω/sq.

The graphene growth using Cu catalyst was also investigated. The Ni capping layer was replaced by a Cu capping layer (200 nm) and the samples were annealed at same growth conditions but at 1000 °C for 4 min because the melting point of Cu (1085 °C) is lower than that of Ni (1455 °C). After annealing, the Raman spectrum of Cu surface did not show any peaks related to carbon (Figure S4a). After Cu etching, the Raman spectrum of the film that remained on the substrate had a large D and G peak without 2D peak; this spectrum is associated with amorphous carbon (Figure S4b). Because the Cu is a surface catalyst due to the low solubility of carbon in it, the Cu layer did not act as an efficient catalyst to form a graphene when our metal capping layer structure was used^43^,^44^.

### Fabrication of graphene-electrode pentacene FETs

**Figure 4:**
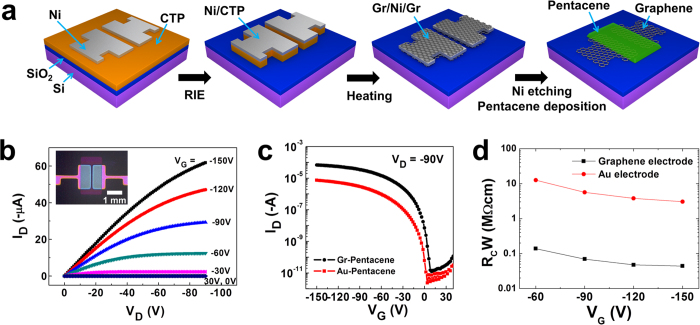
(a) Fabrication process of graphene-electrode pentacene FETs. (**b**) Output characteristics of graphene-electrode pentacene FETs (channel length: 100 μm). Inset: microscopy image of graphene-electrode pentacene FET device. Scale bar: 1 mm. (**c**) Transfer characteristics of graphene-electrode pentacene and Au-electrode pentacene FETs at a fixed V_D_ of −90 V (channel length: 100 μm). (**d**) Contact resistances *R*_*C*_ of graphene and Au electrodes, normalized by channel width *W* (1500 μm).

We used the multi-layer graphene films obtained from our transfer-free method to fabricate bottom-contact OFETs (Fig. 4a) based on graphene electrodes. The patterned graphene electrodes were obtained using shadow evaporation to pattern the metal capping layer. A patterned Ni layer (200 nm) which had a 100-μm gap was deposited using a sputtering system through a shadow mask onto the CTP films. The patterned samples were annealed at 1100 °C for 4 min, which was determined in previous experiments to be the optimal condition for graphene synthesis. However, after annealing, some regions of amorphous carbon occurred where Ni deposition had been prohibited by the shadow mask, especially between the two Ni patterns. Without the Ni capping layer, during the annealing process the CTP film was converted to amorphous carbon film, which is conductive. The amorphous carbon region between the graphene electrodes caused leakage current in the electronic device. After annealing, the Raman spectrum of the gap between the Ni patterns showed a large D and G peaks, which are typical amorphous carbon (Figure S5). Therefore, before annealing we used reactive ion etching (RIE; 100 W, O_2_ gas, 0.2 Torr, 10 s) to remove the CTP film from regions not covered by the Ni pattern. During this process, the patterned Ni layer functions as a passivation mask that protects the CTP film underneath the Ni pattern from RIE treatment. After applying this method, Raman spectrum of the gap between the Ni patterns did not reveal any peaks related to carbon materials (Figure S5); this means that RIE treatment removed the unprotected CTP film effectively. After etching the Ni patterns, the patterned graphene source/drain electrodes (gaps ~100 μm) can be obtained directly on the 500 nm SiO_2_/Si substrate. Finally, a pentacene layer (50 nm thick) was deposited on the patterned graphene electrodes at 50 °C through a shadow mask to complete OFET device fabrication.

**Figure 5:**
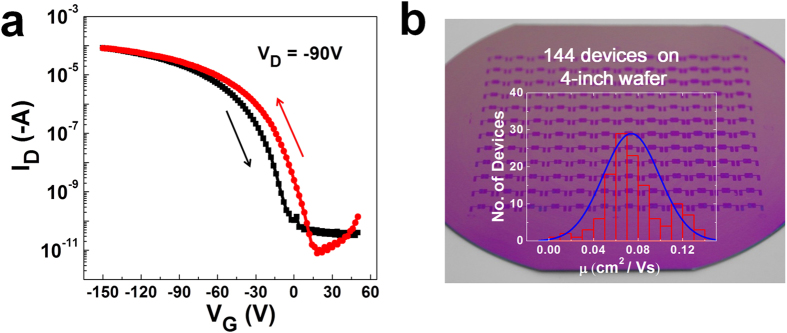
(a) Transfer characteristics of graphene-electrode pentacene FET (red: forward bias, black: reverse bias). (**b**) Photograph of large-area Gr-P FET array of 144 devices on a 4-inch wafer (inset, histogram of the field effect mobility μ_FET_).

The electrical properties of the fabricated bottom contact graphene-electrode pentacene FETs (Gr-P FETs) (Fig. 4b, **inset**) were characterized by measuring their output and transfer characteristics (Fig. 4b,c). For comparison, bottom contact Au-electrode pentacene FETs (Au-P FETs) were fabricated on 500 nm SiO_2_/Si substrates. The output characteristics of the two types of FETs were measured under different linear and saturation current levels (Fig. 4b). The Gr-P FETs showed a clear gating effect and ohmic contact, but Au-P FETs did not have ohmic contact and had low output currents, due to the high contact resistance *R*_*C*_ between Au and pentacene (Figure S6). We calculated *R*_*C*_ of Gr-P and Au-P FETs by using the transfer line method with channel lengths of 30, 50, 80, and 100 μm (Figure S7). As gate voltage varied from −60 to −150 V, *R*_*C*_ of the graphene electrode, normalized by channel width (1500 μm), decreased from 0.14 MΩ·cm to 0.043 MΩ·cm (Fig. 4d), which is about two orders of magnitude lower than that of the Au electrode. This result is consistent with previous reports which demonstrated better FET performance in graphene-electrodes than in common metal electrodes^54^,^55^,^56^,^57^. Gr-P FETs showed transfer characteristics typical of p-type FETs (Fig. 4c). Calculated field-effect mobility *μ*_FET_ in the saturation regime was an order of magnitude higher in Gr-P FETs (0.05–0.13 cm^2^·V^−1^·s^−1^) than in Au-P FETs (0.011–0.017 cm^2^·V^−1^·s^−1^). The transfer curve of the Gr-P FETs showed a high on/off current ratio (1.1 × 10^7^) with small hysteresis (Fig. 5a), so they are suitable for use in circuits and switches of active electronic devices. Our graphene synthesis method enables fabrication of large-area devices; we achieved a large-area Gr-P FETs array of 144 devices on a 4-inch wafer (Fig. 5b**, inset**). In this case, the fabrication process was the same as used to fabricate the Gr-P FETs, except for the use of the large area substrate. The distribution of the μ_FET_ of the Gr-P FET large-area arrays showed ~95% operation and maximum *μ*_FET_ 0.13 cm^2^·V^−1^·s^−1^ (average *μ*_FET_ ~0.07 cm^2^·V^−1^·s^−1^) (Fig. 5b).

## Discussion

**Figure 6:**
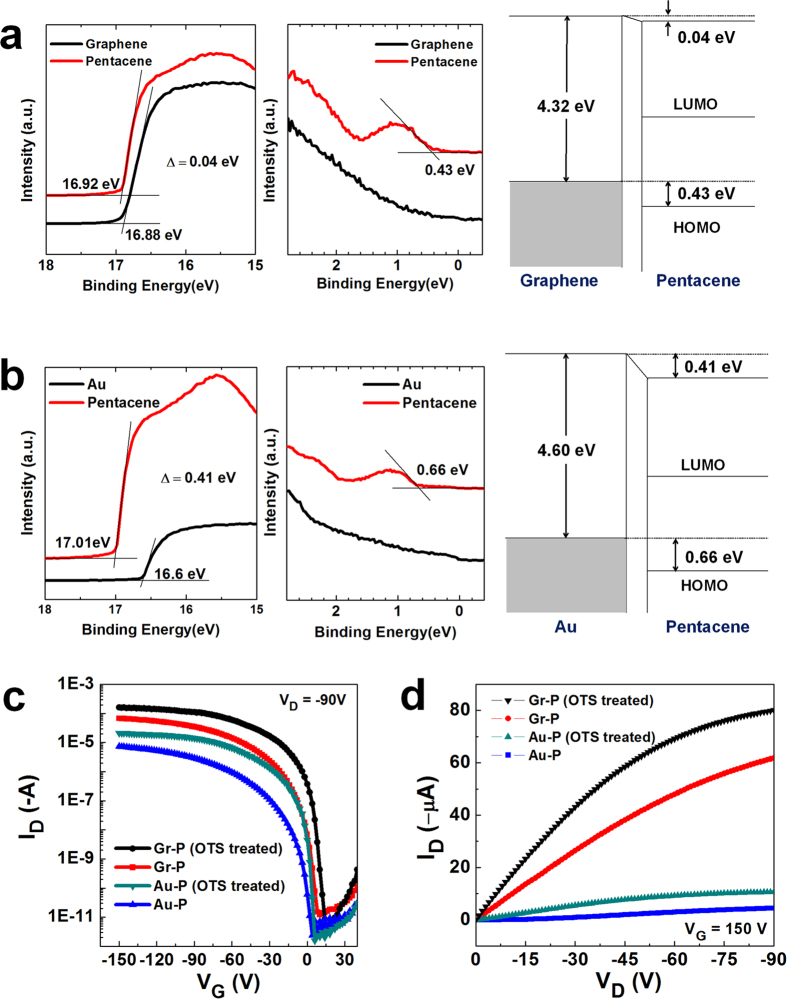
Ultraviolet photoelectron spectroscopy (UPS) spectra and schematic energy diagrams of pentacene on (a) graphene electrode and (b) Au electrode. A 10-nm-thick of pentacene was deposited on graphene and Au electrodes. Lines have been shifted vertically for clarity. The work function of each electrode and hole injection barrier at each interface were estimated from measured UPS values. Lines have been shifted vertically for clarity. (**c**) Transfer characteristics of OTS-treated graphene-electrode pentacene and Au-electrode pentacene FETs at a fixed V_D_ of −90 V (channel length: 100 μm). (**d**) Output characteristics of OTS-treated graphene-electrode pentacene FETs (channel length: 100 μm).

The obvious improvement of electrical properties with Gr-P FETs suggests that the charge injection from graphene to a pentacene layer is superior to that from Au to a pentacene layer due to the low hole injection barriers and low *R*_C_ in Gr-P FETs. *R*_*C*_ between the electrode and the organic channel is primarily affected by the magnitude of the carrier injection barrier at the interface^54^,^55^. The hole injection barrier height at the graphene-pentacene and Au-pentacene interfaces was quantified using ultraviolet photoelectron spectroscopy (UPS) to measure the work function *WF* of each electrode and electrode-pentacene interface. The calculated hole injection barrier of graphene electrode was 0.43 eV, which is 0.23 eV lower than that of Au electrode (0.66 eV), even though the *WF* of the graphene electrode (4.32 eV) was lower than that of Au electrodes (4.60 eV) (Fig. 6a,b). We attribute this result to a difference in interface dipoles between the electrodes and the pentacene layer, which was confirmed by UPS. The graphene-pentacene interface has a small interface dipole (0.04 eV), leading to a small carrier injection barrier; in contrast, the Au-pentacene interface has a large interface dipole (0.41 eV), which resulted in a large vacuum level shift. These differences are the cause of the lower *R*_*C*_ and higher *μ*_FET_ in Gr-FETs compared with the Au-P FETs.

Based on CTP-derived patterned graphene electrodes as an efficient charge injection electrode material, we improved the electrical properties of bottom-contact pentacene OFETs by using surface treatment on dielectric substrates^50^,^51^,^52^,^53^. Treatment using an octadecyltrichlorosilane (OTS) self-assembled monolayer (SAM) has been widely used to enhance ordering of the organic channel and to reduce the interface trapping states^55^,^56^,^57^. Before pentacene deposition, SiO_2_/Si substrates that had graphene and Au electrodes patterns were immersed in an OTS SAM solution (0.1% in toluene) for 3 min, then dried under blowing using N_2_ gas. The OTS-treated Gr-FETs showed increased drain current and μ_FET_ (0.25–0.36 cm^2^·V^−1^·s^−1^), and the OTS-treated Au-FETs were also affected (μ_FET_ 0.04–0.07 cm^2^·V^−1^·s^−1^) (Fig. 6c,d).

In conclusion, we demonstrated a simple, scalable, low-cost, ecologically sustainable, value-added process to synthesize graphene films from inexpensive CTP films under a Ni capping layer without using explosive hydrocarbon gas sources and physical transfer process. We synthesized multi-layer graphene films underneath the patterned Ni layer in a tube furnace and then achieved patterned graphene films as source/drain electrodes directly on the OFET device substrate without physical transfer process after removing the patterned Ni layer. The pentacene FETs using graphene source/drain electrodes synthesized using our transfer-free patterned growth method showed higher carrier mobility (max. *μ*_FET,_ ~0.36 cm^2^·V^−1^·s^−1^) in a bottom-contact geometry than did Au-electrode pentacene FETs. Using the same method, we also fabricated large-area graphene-electrode pentacene FET arrays (144 devices on a 4-inch wafer) to demonstrate transfer-free fabrication of graphene-based electronic devices. Our results provide a promising way to use graphene films converted from the carbon waste (CTP) as a reliable conducting material and as an alternative electrode for electronic devices. Furthermore, using this industrial waste product of steelworks to produce graphene will contribute to a clean environment and sustainable graphene growth.

## Methods

### Synthesis of Graphene from coal tar pitch

The coal tar pitch (SP = 60.8 °C, RIST) was diluted with quinoline solvent (JUNSEI Chemical) to a concentration of 8 wt%. The Coal Tar Pitch solution was spin coated (6000 rpm, 60 s) on a SiO_2_ (500 nm)/Si substrates (NAMKANG HI-TECH). After baking at 240 °C for 30 min, the Ni layer (200 nm) was deposited (Magnetron Sputtering System, SNTEK; working pressure 7 mTorr; power 50 W; Ar flow rate 50 sccm) on a coal tar pitch film at room temperature. Then the sample was annealed using thermal CVD (1100 °C, 4 min) with flowing 50 sccm Ar and 10 sccm H_2_ at a total pressure of ~0.3 Torr, then cooled to room temperature by removing the tube from the furnace while maintaining the same Ar/H_2_ flow. The Ni layer was etched away by dipping the sample into FeCl_3_ solution (Iron(III) chloride solution 45°Be’, JUNSEI Chemical) for 1 min and the sample was rinsed with DI water. Raman spectra and mapping images of graphene films were investigated using a Raman spectroscopy (WITEC Alpha 300R Raman spectroscope equipped with a 532 nm diode laser). The CTP-derived graphene was further characterized by HR-TEM (Titan Cubed Themis, 80 keV).

### Graphene and Au-electrode pentacene FETs

A 50-nm-thick pentacene (Sigma Aldrich) layer was deposited on the electrodes through a shadow mask at 50 °C. The electrical properties of the graphene and Au-electrode pentacene FETs were characterized using a parameter analyzer (Keithley 4200). The work function of each electrode and electrode-pentacene interface was measured using ultraviolet photoelectron spectroscopy (UPS) (AXIS-NOVA, Kratos. Inc).

## Additional Information

**How to cite this article**: Seo, H.-K. *et al.* Value-added Synthesis of Graphene: Recycling Industrial Carbon Waste into Electrodes for High-Performance Electronic Devices. *Sci. Rep.* **5**, 16710; doi: 10.1038/srep16710 (2015).

## Supplementary Material

Supplementary Information
