# Supplementary material for: Value-added Synthesis of Graphene: Recycling Industrial Carbon Waste into Electrodes for High-Performance Electronic Devices
Source: Sci Rep. 2015 Nov 16;5:16710. doi: 10.1038/srep16710 (PMC4644944; doi:10.1038/srep16710)
Supplement: Supplementary Information [file srep16710-s1.pdf]

Supporting Information

Value-added Synthesis of Graphene: Recycling Industrial Carbon Waste into Electrodes for High-Performance Electronic Devices

Hong-Kyu Seo[1], Tae-Sik Kim[1], Chibeom Park[2], Wentao Xu[1], Kangkyun Baek[3], Sang-Hoon Bae[4], Jong-Hyun Ahn[4], Kimoon Kim[3,5], Hee Cheul Choi[2,5], and Tae-Woo Lee[1,5,*]

[1] Department of Materials Science and Engineering, Pohang University of Science and Technology (POSTECH), Pohang, Gyungbuk 790-784, Republic of Korea
[2] Department of Chemistry, Pohang University of Science and Technology (POSTECH) and Center for Artificial Low Dimensional Electronic Systems (CALDES), Institute for Basic Science (IBS), Pohang, Gyungbuk 790-784, Republic of Korea
[3] Department of Chemistry, Pohang University of Science and Technology (POSTECH) and Center for Self-assembly and Complexity (CSC), Institute for Basic Science (IBS), Pohang, Gyungbuk 790-784, Republic of Korea
[4] School of Electrical and Electronic Engineering, Yonsei University, Seoul 120-749, Republic of Korea
[5] Division of Advanced Materials Science, Pohang University of Science and Technology, Pohang, Gyungbuk 790-784, Republic of Korea
[*] twlee@postech.ac.kr; taewlees@gmail.com

Table S1. Ellipsometry measurements of thickness of CTP films from CTP with various softening points. Film uniformity was confirmed by atomic force microscopy (AFM).

Softening Points (℃) of CTP	60.8 ℃	82.2 ℃	116.7 ℃	167.7 ℃
Film Thickness (nm)	20 nm	25 nm	30 nm	40 nm
Roughness (RMS)	0.294 nm	0.288 nm	0.290 nm	0.285 nm
AFM Image (5㎛ x 5㎛)	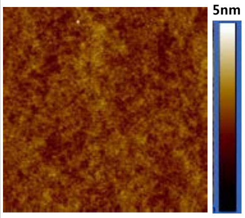	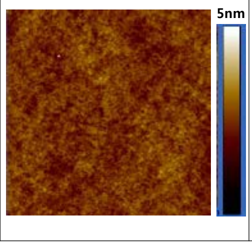	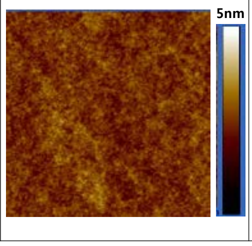	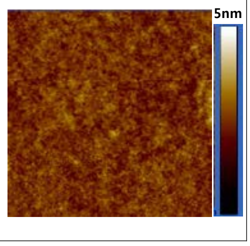

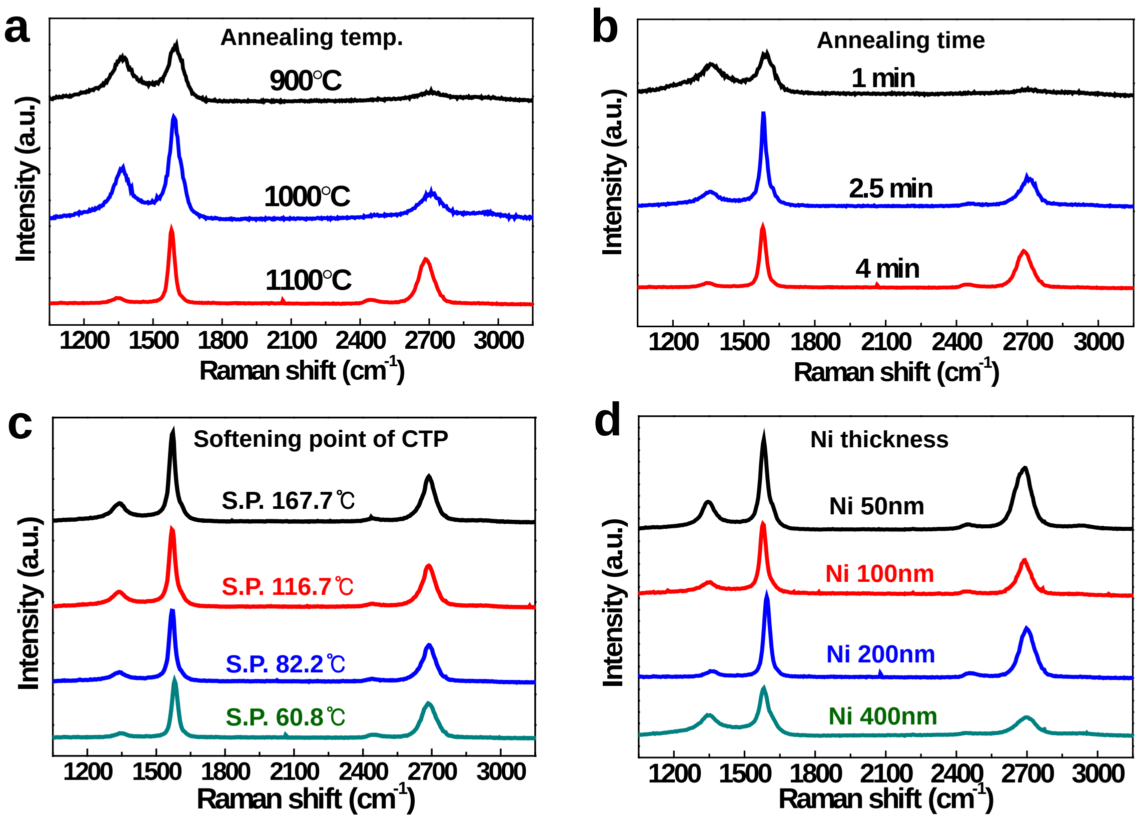

Figure S1. Raman spectra of coal tar pitch-derived graphene grown on Ni surface after annealing. (a) Raman spectra of graphene depending on the annealing temperature for 4 min. (b) Raman spectra of graphene depending on the annealing time at 1100 °C. (c) Raman spectra of graphene depending on the softening point of coal tar pitch at 1100 °C for 4 min. (d) Raman spectra of graphene depending on Ni layer thickness at 1100 °C for 4 min. Lines have been shifted vertically for clarity.

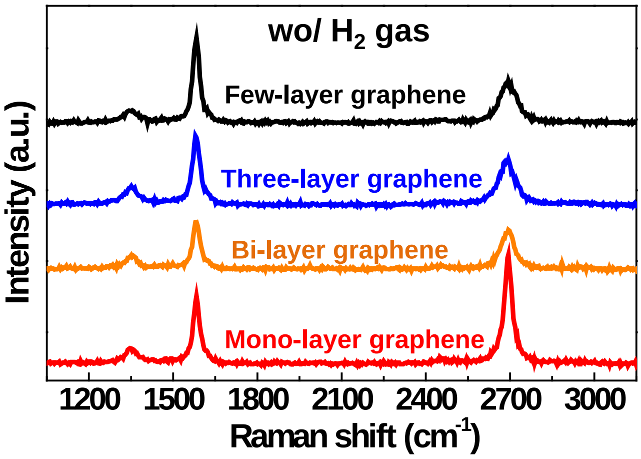

Figure S2. Raman spectra of graphene at different points of sample from mono-layer to few-layer from the H2-gas-free synthesis. Lines have been shifted vertically for clarity.

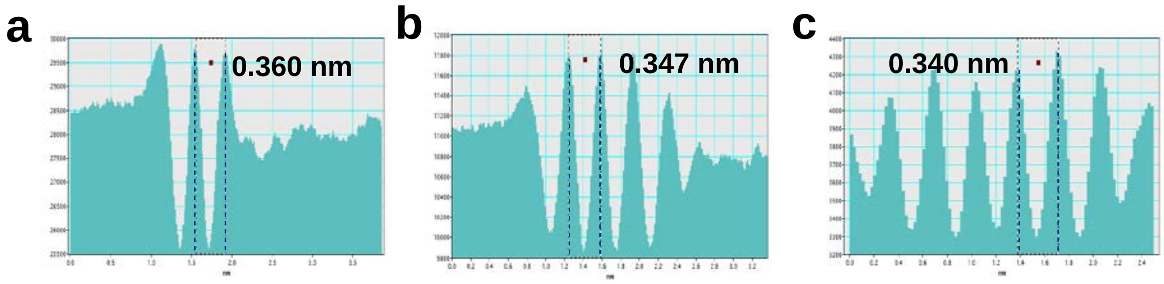

Figure S3. Intensity profiles from TEM images which show the inter layer distance. (a) 2 layers (b) 3-4 layers (c) > 5 layer

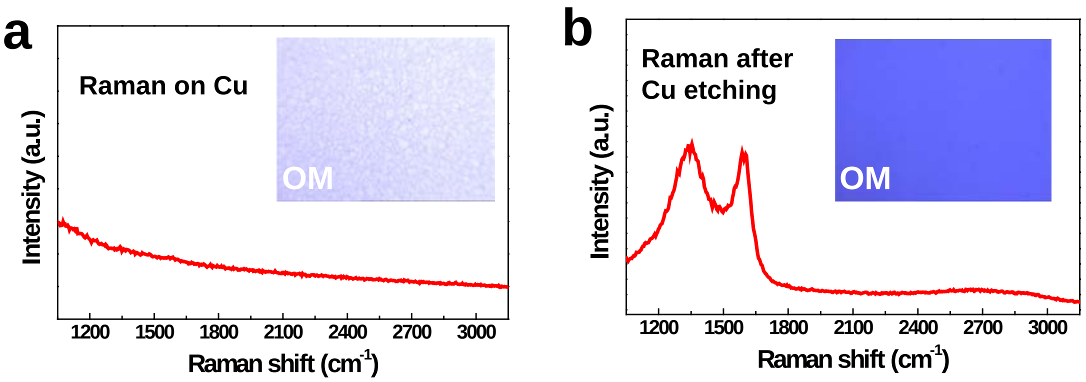

Figure S4. (a) Raman spectrum of Cu surface after annealing. (b) Raman spectrum from substrate after Cu etching. (inset, optical microscope image)

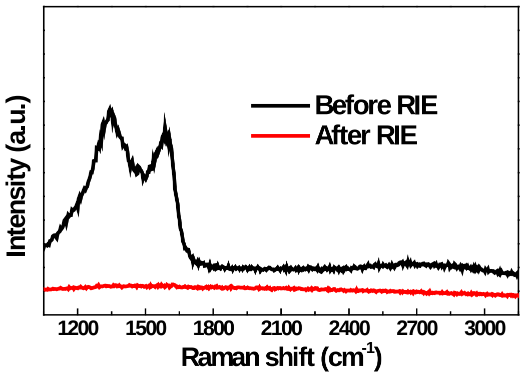

Figure S5. Raman spectra of the gap between graphene electrode patterns before (black) and after (red) RIE.

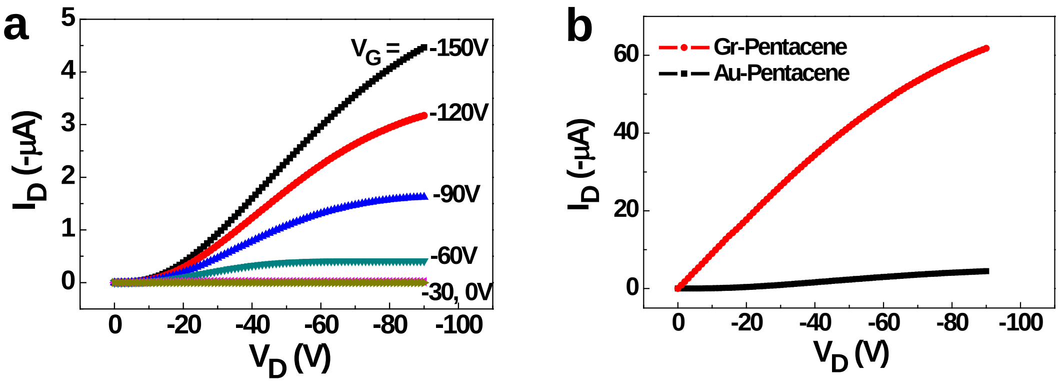

Figure S6. (a) Effect of gate voltage V_G on drain current I_D vs drain voltage V_D of Au-electrode pentacene FETs (channel length: 100 μm). (b) I_D vs V_D of graphene-electrode pentacene and Au-electrode pentacene FETs at V_G = -150 V (channel length: 100 μm).

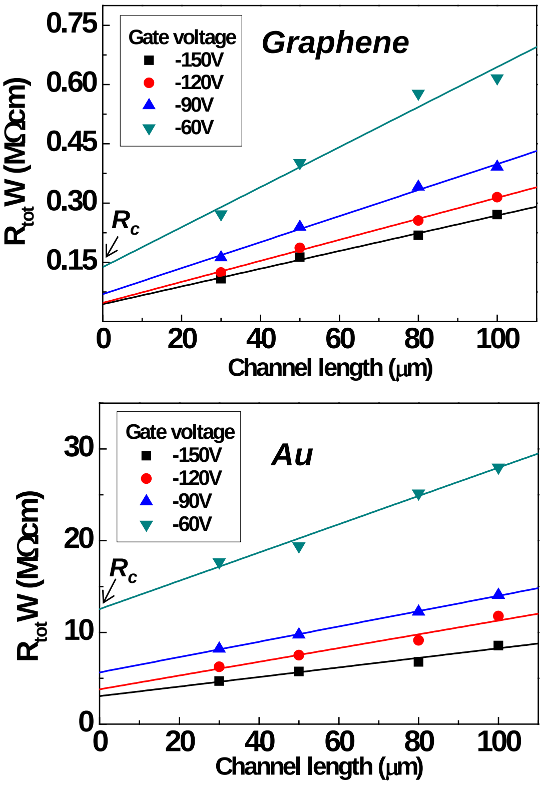

Figure S7. Channel-width-normalized total resistances R_{tot} of pentacene FETs with (a) graphene electrodes, and (b) Au electrodes. The contact resistance R_C of Gr-P and Au-P FET at each gate voltage was calculated from the L = 0 intersection of R_{tot} with channel lengths L of 30, 50, 80, and 100 μm. These channels were fabricated by photolithographic techniques and the devices were fabricated on bare SiO_2 substrates without any surface treatment.
